# Can granivorous birds disperse stick and leaf insect eggs? Integrating in vivo digestion trials and in vitro functional experiments

**DOI:** 10.1007/s00114-026-02157-5

**Published:** 2026-09-11

**Authors:** Judith Burack, Eberhard Haase, Stanislav N. Gorb, Thies H. Büscher

**Affiliations:** Functional Morphology and Biomechanics, Institute of Zoology, Kiel University, Kiel, Germany

**Keywords:** Egg dispersal, Egg survival, Mechanical digestion, Ornithochory, Phasmatodea, Insect

## Abstract

Spatial distribution plays a crucial role in speciation, affecting genetic divergence and reproductive isolation. Phasmatodea (stick and leaf insects) have limited adult mobility and may use their seed-like eggs for passive dispersal. The possibility of egg dispersal through birds is controversially discussed in current literature. Previous studies suggest that ornithochory of phasmid eggs is context-dependent and most eggs do not survive digestion by granivorous birds, while eggs consumed by insectivorous birds can potentially survive and hatch, presumably also when a gravid female is consumed. This study examined this question by testing how different phasmid egg morphologies withstand bird digestion, simulating the digestive system in laboratory conditions and evaluating egg viability post-treatment. Results showed that none of the eggs of the tested species hatched after being eaten by granivorous birds, although some eggs of the species *Dajaca napolovi* were found structurally intact. In the laboratory experiments, the low pH environment significantly affected survival, with none of the treated eggs surviving acid treatment. High temperatures and mechanical impact also influenced egg hatching depending on the species. The findings suggest that acid and temperature tolerance are prerequisites for bird-mediated dispersal of single eggs ingested by granivorous birds and mechanical impact by the gizzard renders long-distance dispersal via ornithochory by granivorous birds unlikely for the investigated phasmatodean species. To further elucidate the potential ornithochory of phasmid eggs by birds with different diets, studies examining the digestive conditions experienced by ingested eggs in other bird species are warranted, particularly regarding insectivorous birds and the role of potential protection of eggs within an adult female phasmid.

**Supplementary Information:**

The online version contains supplementary material available at https://doi.org/10.1007/s00114-026-02157-5.

## Introduction

Geographic isolation significantly influences species formation through processes, such as genetic divergence, reproductive isolation, and the distribution of organisms across various environments (Suetsugu et al. 2023; Mayr 1942; Haffer 1969). The dispersion of species occurs not only for exploring new locations but also for preventing inbreeding and interspecific competition (Clobert 2012; Keller 2002; O’Hanlon et al. 2020). Species with limited mobility employ different ways to reach relatively distant areas. Some travel further by attaching to highly mobile animals (Büscher et al. 2022; Preuss et al. 2024) or dispersing during the embryonic stage of their life cycle (Johnson and Gaines 1990; Lovas-Kiss et al. 2020).

Plants, as sessile organisms that primarily rely on their seeds for dispersal, exhibit various strategies for reaching new locations. For instance, some plants have a catapult mechanism to propel their seeds over long distances or depend on frugivores for dispersal. Frugivores consume the seeds along with their fleshy outer layer and later excrete them, often at a different location from the mother plant (Docters van Leeuwen 1954; Howe, 1986; Kreitschitz et al. 2024; Traveset et al. 2014). Other plants have developed mutualistic interactions with ants: myrmecochory. The ants carry the seeds into their nests, protecting them from fire, granivores, and other threats that could endanger the seeds above ground. In return, the ants receive the elaiosome, a lipid-rich appendage that can be removed without harm from the seed (Gorb and Gorb 2003; Hanzawa et al. 1988; Boyd 2001).

Phasmids, herbivorous insects that mimic parts of plants (Bedford 1978), exhibit limited self-distribution in their adult forms. While many insects use wings to travel greater distances, in phasmids wings are often reduced or entirely absent (Bank and Bradler 2022; Bradler 2015; Compton and Ware 1991; Forni et al. 2022; Whiting et al. 2003; Zeng et al. 2023). Sometimes, even when functional wings are present, they are hardly used (Maginnis 2006). Moreover, not only do the adult individuals resemble parts of plants, but their eggs also resemble plant seeds (O’Hanlon et al. 2020; Bedford 1978; Sharp, 1898; Severin 1910). It has been suggested that the mimicry of plant seeds may serve as protection against egg parasites and ovivores, although most of them rely more on chemical stimuli than visual cues (O’Hanlon et al. 2020; Severin 1910; Göldi 1886; Grimpe 1921). The oviposition strategies of phasmids can be categorised into two main types: placed and unplaced oviposition (Carlberg 1983). Placed oviposition includes strategies, such as glueing eggs to plant surfaces or burying them in the ground. Flicking or simply dropping the eggs is regarded as unplaced oviposition. Particularly for unplaced eggs, the eggs of phasmids resemble plant seeds in appearance and dispersal method (O’Hanlon et al. 2020).

The outer layers of phasmid eggs contain a high mineral content, which imparts rigidity and makes them resistant to impacts from surrounding chemical and mechanical influences (Robertson et al. 2018; Moscona 1950; Kobayashi et al. 2014). Depending on the species, the outer egg consists of multiple layers with varying compositions (Moscona 1950; Brock and Büscher, 2022; Debruyn et al. 2025; Leuzinger et al. 1926). One of these layers contains biomineralised material, including calcium oxalate, which appears to dissolve partially in low pH environments (Moscona 1950; Debruyn et al. 2025; Leuzinger et al. 1926). Some phasmid eggs also display hairlike structures on their surface or other features resembling plant seeds (Büscher et al. 2024). Several phasmid eggs have a capitulum, a lipid-rich structure on top of the operculum, which not only visually resembles the elaiosome of plant seeds but also exhibits similar chemical features and responses from ants (Compton and Ware 1991; Hughes and Westoby 1992; Stanton et al. 2015).

Given the similarities in appearance between phasmid eggs and seeds, the higher risk of being eaten by granivorous birds must also be considered (Göldi 1886). A study by Shelomi (Shelomi 2011) investigated whether the eggs of *Extatosoma tiaratum*, *Ramulus nematodes*, and *Ramulus artemis* could survive being eaten by chickens and thereby be dispersed over longer distances via birds. Only one egg of *E. tiaratum* remained undamaged. Every other egg was digested beyond recognition, suggesting that dispersal through the digestive system of granivorous birds was quite unlikely (Shelomi 2011). Nevertheless, in a different context ornithochory might be realised by different vectors. In another study excised eggs from gravid females of three phasmid species fed to insectivorous brown-eared bulbuls resulted in a few eggs of *Ramulus mikado* (syn. *Ramulus irregulariterdentatus)* still hatching (Suetsugu et al. 2018). Due to the facultative parthenogenesis of some phasmids, the eggs within a female phasmid can also be viable (Suetsugu et al. 2018; Suomalainen 1962), potentially rendering ingestion of gravid females a possible route for ornithochory. Suetsugu also investigated the phylogeography of *Ramulus mikado* for evidence of long-distance dispersal through birds and proposed that it might be possible under such conditions (Suetsugu et al. 2023).

Whether phasmid eggs can survive passage through the avian digestive tract and consequently be dispersed by birds likely depends on the ecological and physiological context. Nevertheless, the functional mechanisms underlying this phenomenon remain poorly understood. Firstly, bird-mediated dispersal was so far studied mainly considering mechanical intactness of the egg for granivorous birds (Shelomi 2011) or hatching of the embryo for insectivorous birds (Suetsugu et al. 2018). Secondly, varying adaptations favouring survival of eggs during bird digestion across species in Phasmatodea, as well as the predominant parameters in the birds’ digestive system besides mechanical abrasion through the gizzard, were not assessed. Consequently, the parameters that actually impair egg survival of the birds’ digestion remain speculative as well as the adaptations that foster it. This study aims to identify these factors by examining how different egg morphologies affect the morphological integrity and survival of the eggs during the passage of the digestive system of granivorous birds. Therefore, we tested the morphologically diverse eggs from seven phasmid species being consumed by pigeons and additionally emulated the different parameters of the general digestive system of birds in laboratory conditions, assessing the eggs' viability after these treatments.

## Material and methods

**Fig. 1:**
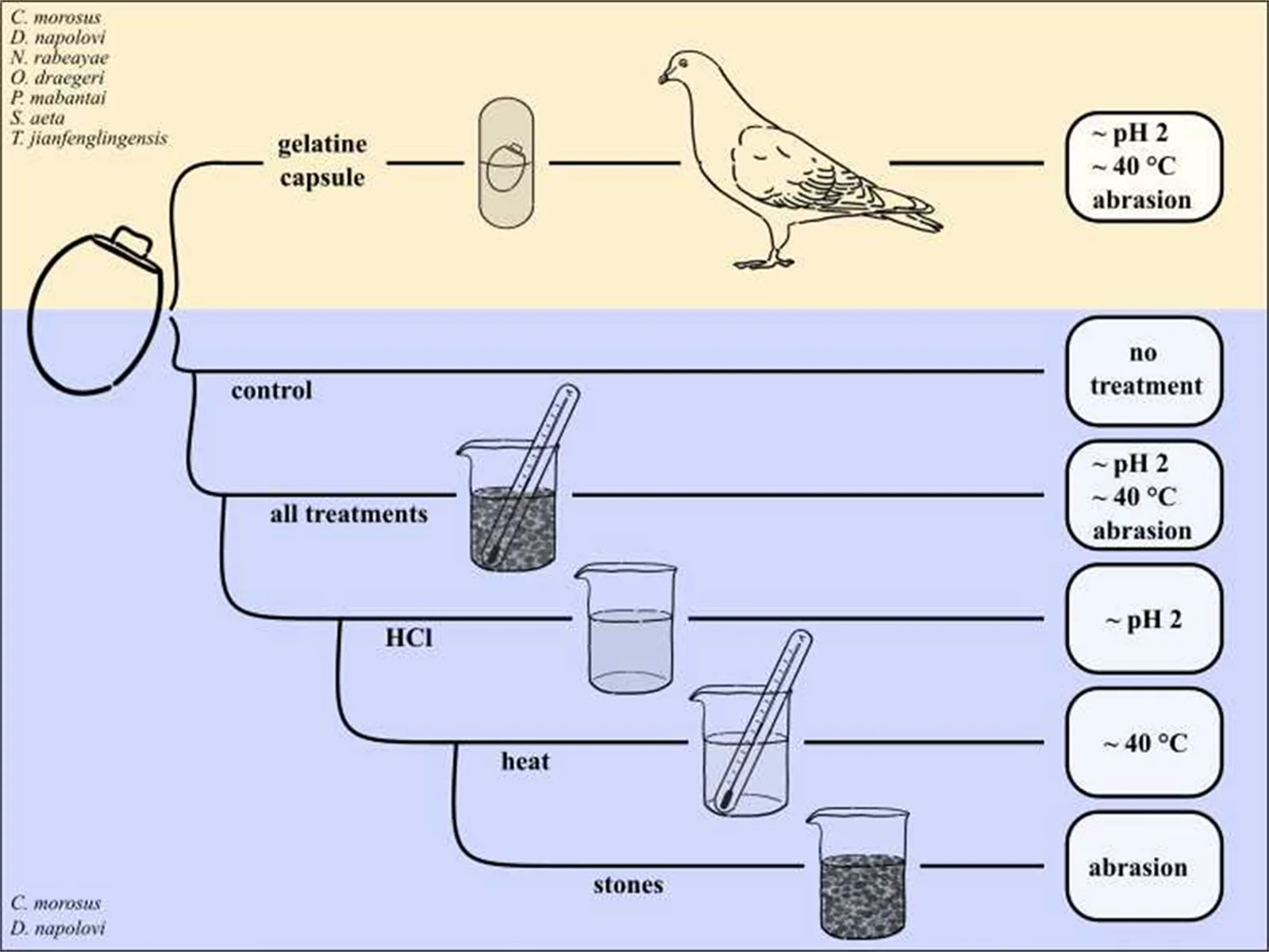
Overview of the experiments performed in this study. The upper part shows the feeding of the phasmid eggs to a pigeon, and the lower part the experiment of the imitation of the bird’s digestive system

Two different approaches to examine the endozoochoric potential of phasmids through birds were performed in this study: feeding eggs to birds and treating eggs with emulated conditions of the digestive system of birds in the laboratory (Fig. 1). The phasmid eggs used in this study were freshly collected from animals of laboratory cultures and all checked visually for intactness.

### Feeding experiments with pigeons

**Fig. 2:**
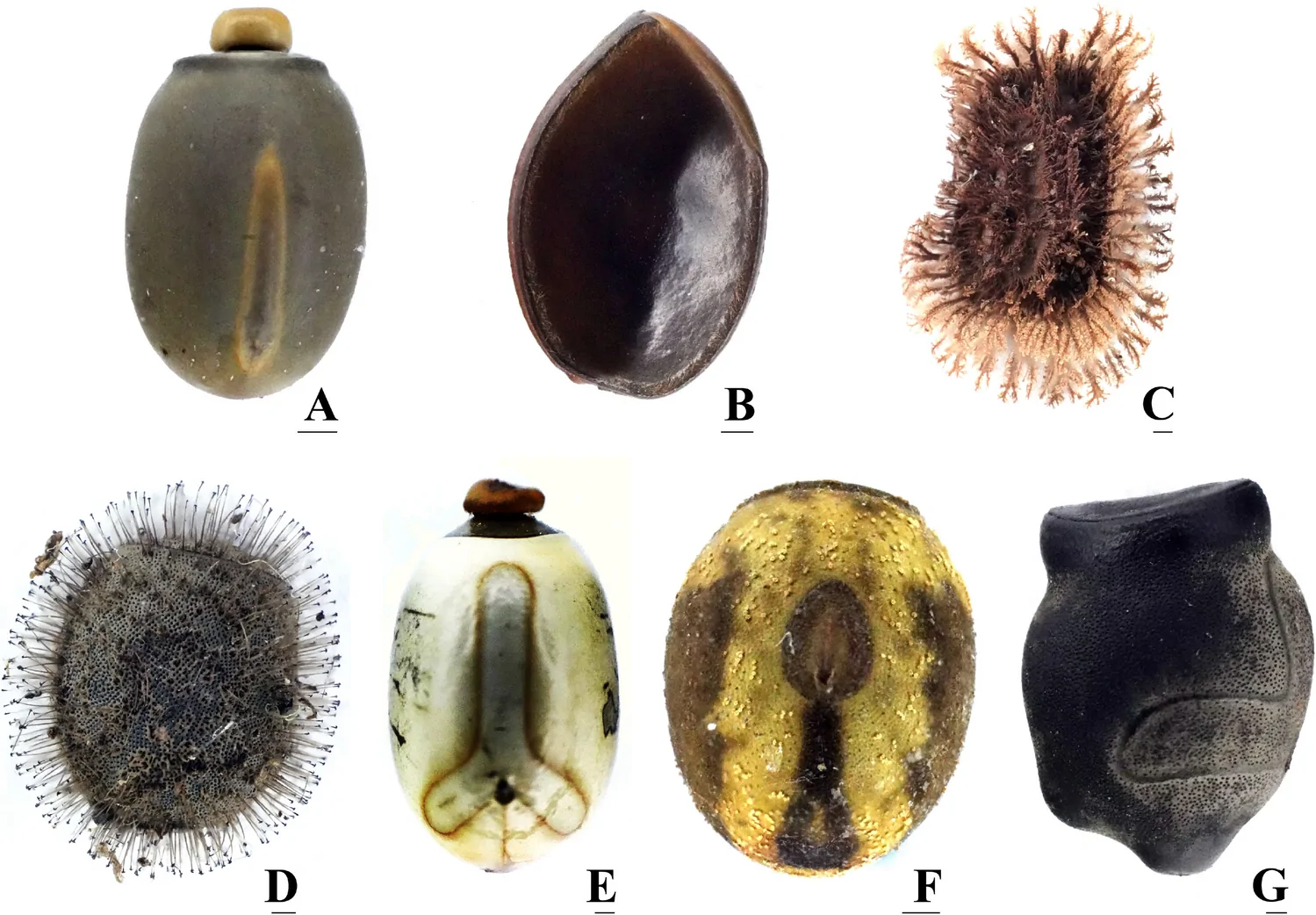
Phasmid eggs used in this study. **A** *Carausius morosus*. **B** *Dajaca napolovi*. **C** *Phyllium mabantai.* **D** *Orestes draegeri*. **E** *Tirachoidea jianfenglingensis*. **F** *Nuichua rabaeyae*. **G** *Sungaya aeta*. Scale bars: 250 µm

To investigate whether phasmid eggs could survive being eaten by a bird, eggs of different phasmid species were fed to pigeons (*Columba livia* f. *domestica*). For this purpose 50 eggs each of the species *Carausius morosus* (Brunner, 1907) (Phasmatidae), *Dajaca napolovi* Brock, 2000 (Aschiphasmatidae), *Nuichua rabaeyae* Bresseel & Constant 2018 (Lonchodidae), *Orestes draegeri* Bresseel & Constant 2018 (Heteropterygidae), *Phyllium mabantai* Bresseel, Hennemann, Conle & Gottardo, 2009 (Phylliidae), *Sungaya aeta* Hennemann, 2023 (Heteropterygidae) and *Tirachoidea jianfenglingensis* (Bi, 1994) (Phasmatidae) were used (Fig. 2). The species were selected to account for different morphotypes across phasmid eggs (Büscher et al. 2024) and include capitulate small (Fig. 2A) and large (Fig. 2E) eggs, as well as lentil-shaped (Fig. 2B), pinnate (Fig. 2C), hairy (Fig. 2D), uncapitulate eggs with rough surface patterns (Fig. 2F) and eggs that are buried with a secondary ovipositor (Fig. 2G). The eggs of each species were inserted in amounts of 16–25 eggs into a 1 mL gelatine capsule (Gelatine-Kapseln C 9 VE, EYDAM, Kiel, Germany) and fed to the pigeons in addition to their normal seed mix. The droppings of the pigeon were collected on a paper sheet and carefully investigated for eggs and egg pieces. The droppings were dissolved in distilled water and egg fragments were manually sorted from the bird excrements. Findings were examined using a light microscope (M205, Leica Microsystems Ltd., Wetzlar, Germany), pictures taken with a Sony RX10 camera (Sony Corporation, Tokyo, Japan) and edited with Adobe Photoshop CS4 (Adobe Inc., San Jose, United States). Intact eggs were incubated in a Petri dish lined with damp paper towel under normal room conditions (21–24 °C, 49–61% ambient humidity) and monitored on a daily basis.

### Pigeon husbandry

The racing pigeons used were from a private colony and treated as described in (Kreitschitz et al. 2021). Adult pigeons (age > 1 year) were fed with eggs in their familiar environment. Birds were housed in a room of a wooden garden house (dimensions: 3.3 m × 2.0 m 2.5 m (length x width x height) with two windows). The pigeons were released twice a day, in the morning and in the afternoon, for free flight sessions of 30–60 min, after which they were called back into their loft. Prior to the experiment, the birds were fed their normal diet and water. About 30 min later, each specimen was given a slightly moistened 1‑ml gelatine capsule filled with the eggs, which was placed deep into the throat to ensure swallowing. Subsequently, the pigeons were housed individually for 24 h in lockable nest boxes (35 × 49 cm floor area, 39 cm height) without access to food or water. The boxes remained undisturbed during this period. Each box was equipped with a plastic‑coated wire mesh grid positioned above a paper sheet covering the floor, so that droppings would fall onto the paper. After 24 h, the paper sheets with the collected droppings were immediately transferred to the laboratory for analysis, and the pigeons were released from the boxes.

### Imitation of a bird digestive system

To simulate the conditions of a bird digestive system, an experimental setup similar to the one used by (Kreitschitz et al. 2021) for plant seeds was applied to the phasmid eggs in this study. In total, 250 eggs of *Carausius morosus* and 250 eggs of *Dajaca napolovi* were used. The pH value, body temperature and mechanical stress which an egg is exposed to within the birds’ digestive system (Ziswiler et al. 1972) were tested separately and with all parameters combined (Fig. 1). For each condition, a plastic laboratory container or a glass measuring cup with 50 eggs of each species and distilled water were put onto a magnetic stirrer (2mag, hotMIX, Germany) and mixed for 1 h. The pH of 1.9–2.2 was generated by using 10 ml of 0.01 M HCl instead of distilled water. To mimic the body temperature of the bird, the water was heated to 38–42 °C. For emulating the mechanical stress of the bird gizzard small stones of 2–3 mm in size were stirred with the eggs and distilled water.

After one hour of treatment, the eggs were washed with distilled water and placed on a Petri dish lined with a damp paper towel. As a control, 50 untreated eggs for either species were placed in Petri dishes as well. To investigate the hatching ability of the treated eggs, the Petri dish was kept in the laboratory under normal conditions (21–24 °C, 49–61% ambient humidity) and the paper towel was kept in a damp state being rewatered every 2–3 days. The incubation Petri dishes were monitored on a daily basis. If an egg hatched, the phasmid and the egg shell were removed. The eggs were incubated in this condition until no further hatching of eggs was occurring. Incubation time was calculated from the noted oviposition date. For *C. morosus,* the eggs included in the groups of 50 per treatment were oviposited on the same day, however, because of the limited number of adult egg-laying females of *D. napolovi,* the sets of 50 eggs used in the assay were collected over a range of 3–4 days. For the calculation of the incubation time, the last day of collecting was considered for this species.

The treated eggs were analysed optically with a light microscope (Olympus SZX12) and a Hitachi TM3000 scanning electron microscope (Hitachi High-Technologies Corp., Tokyo, Japan). Images were taken with a camera (Sony RX10) at a light microscope and edited with Photoshop CS4. The scanning electron microscope specimens were sputter-coated with a 10 nm thick gold–palladium film using a Leica EM SCD 500 High Vacuum Sputter Coater (Leica Microsystems GmbH, Wetzlar, Germany).

### Friction performance

The friction behaviour of the eggs of *Carausius morosus* and *Dajaca napolovi* was assessed using a motorized tilting stage as used in previous studies for seed friction measurements (Gorges et al. 2025; Kreitschitz et al. 2015). This was done to quantify the likelihood of eggs to survive the passage of the bird gizzard due to their slipping ability between the stones in the gizzard. For this purpose, sets of four eggs were glued on a microscope slide (76 × 26 mm; Thermo scientific, Budapest, Hungary) to ensure sliding of the egg surface and prevent tilting during the experiments. In total, 25 sets of eggs of *C. morosus* (*N* = 100, *m* = 4.52 ± 0.77 mg) and 12 sets of *D. napolovi* (*N* = 48, *m* = 4.67 ± 0.49 mg) were used. Epoxy resin casts (Low Viscosity Spurr Kit, Structure Probe Inc. West Chester, PA, USA) with different roughness were mounted on the tilting platform and the microscope slides were placed on them with the eggs in contact with the epoxy substrate. The platform was then tilted at an angular velocity of 3.5° per second until the eggs started sliding. At onset of sliding, the platform was stopped and its angle (*α*) was determined. The friction coefficient (*µ*) of the egg-substrate pairs was determined following:

$$\mu\;=\;\mathrm{tan}\;\alpha$$

The epoxy substrates were replicas of glass (0 µm roughness) and different polishing papers (1 µm, 12 µm and 40 µm roughness). For the production, profilometric and water contact angle data of these substrates see (Büscher et al. 2023).

### Statistics

The influence of the emulated bird digestive system experiments was tested using chi-squared tests. The friction performance was compared using a Generalized Linear Model with species and substrate as independent and friction coefficient as dependent variable. The data were tested for normality (Shapiro–Wilk) and homogeneity of variance (Levene). The Holm-Šídák test was used as post hoc test. Statistics were done using SigmaPlot 12.5 (Systat Software, San Jose, CA, USA).

## Results

### Feeding experiments

**Fig. 3:**
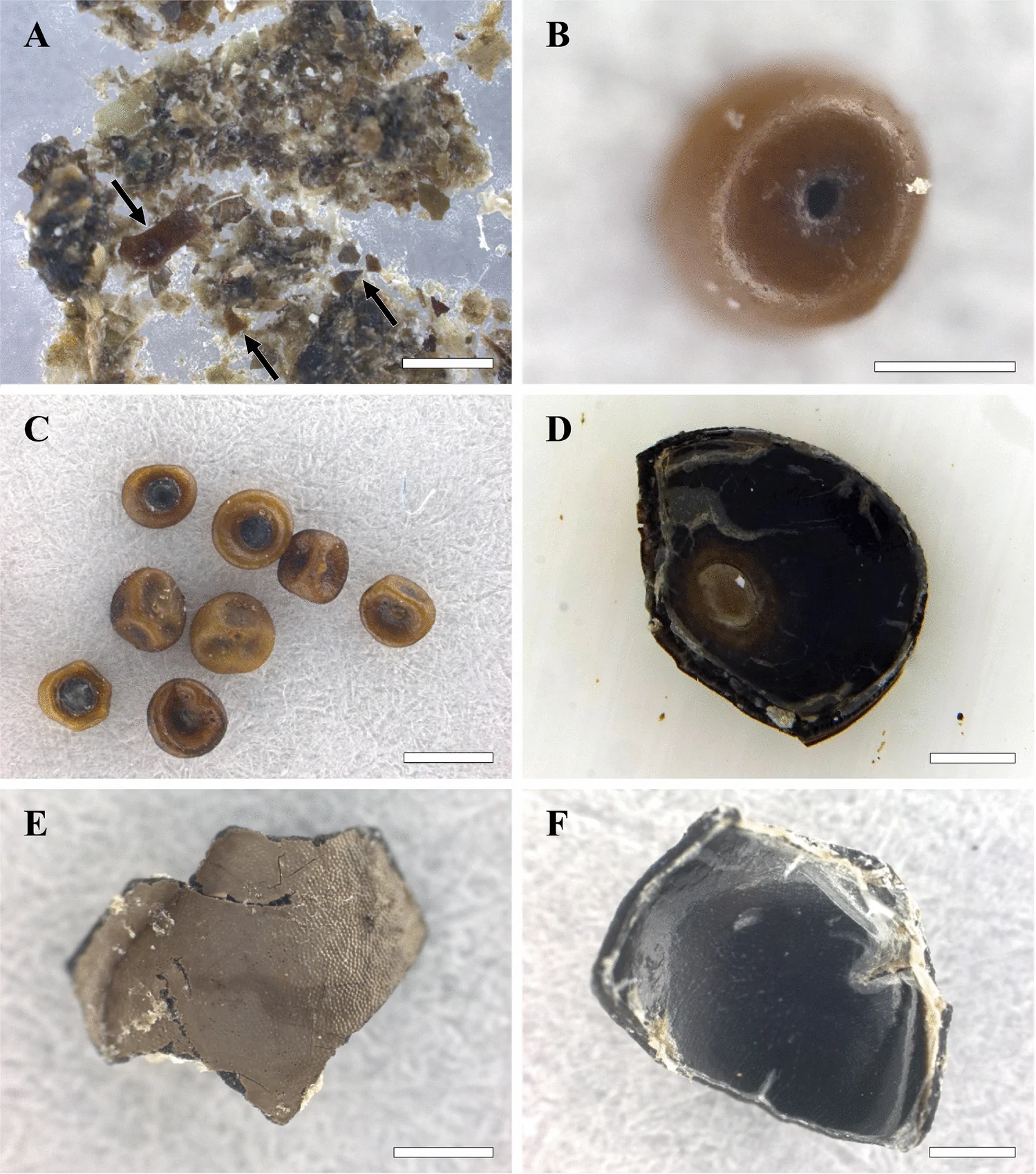
Egg pieces found in the pigeon droppings. Pictures were taken through a light microscope with a Sony RX10 camera. **A** Bird droppings. Arrows mark pieces of eggshells. **B** Capitulum of *Carausius morosus*. **C** Capitula of *Tirachoidea jianfenglingensis.* **D** Fractured operculum of *T. jianfenglingensis.* **E** Eggshell fragment of *Sungaya aeta.* **F** Eggshell fragment of *T. jianfenglingensis*. Scale bars: A, C: 2 mm, B: 500 µm and D–F: 1 mm

In the pigeon droppings, eight intact eggs of *D. napolovi* and some egg pieces were found (Fig. 3). Most eggs were ground to very small pieces. The larger detected pieces were mostly fractured opercula of the species *C. morosus* (Fig. 3B) and *T. jianfenglingensis* (Fig. 3C and D). Larger pieces of eggs were only found for the species *T. jianfenglingensis* and *S. aeta*. Interestingly, capitula were frequently found fully intact (Fig. 3C). None of the morphologically intact eggs that passed through the pigeon digestive system hatched afterwards.

### Results of the emulated bird digestive system assay

**Fig. 4:**
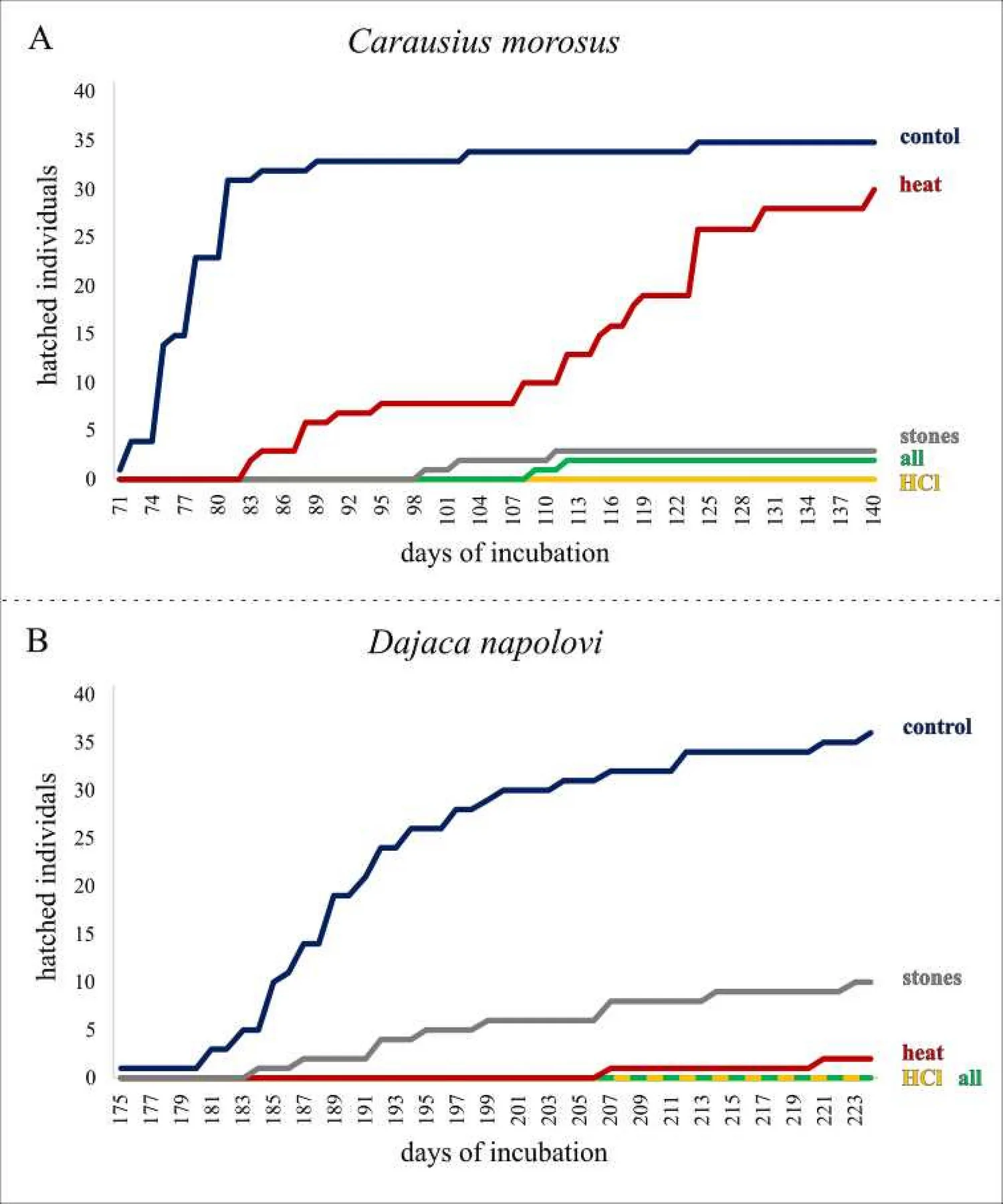
Incubation time of the eggs. **A** *Carausius morosus*, **B** *Dajaca napolovi* over the time of incubation

For each influencing parameter of the digestive system of birds (acidity, temperature, and mechanical stress), the combined treatment and a control group for each species 50 eggs of *C. morosus* and *D. napolovi* were tested. Data for hatching duration across treatments and species are included in the supplementary Table 1. The highest number of eggs hatched for both species within the control group (Fig. 4).

In the control group of *C. morosus* 35 of 50 eggs (70%) hatched with a mean incubation time of 79.6 ± 2.8 days (mean ± SD). The second-highest number of *C. morosus* eggs hatched at the heat treatment with 30 of 50 hatched eggs (60%). Those eggs showed a mean incubation time of 112.0 ± 1.6 days. Nearly all the *C. morosus* eggs stirred together with stones were crushed but of the undamaged eggs three hatched with a mean incubation time of 104.0 ± 0.3 days. Two eggs hatched out of the 50 eggs treated with all factors. The eggs hatched on days 110 and 112 of incubation (110.5 ± 0.3 days). However, none of the *C. morosus* eggs exposed to low pH hatched. The influence of the treatment on the hatching time was statistically significant (*χ*^*2*^ = 114.881, d.f. = 4, *p* ≤ 0.001).

Compared with *C. morosus,* fewer eggs of *D. napolovi* hatched in total. In the control group 36 of 50 *D. napolovi* eggs hatched (72%). Those eggs had a mean incubation time of 192.7 ± 1.4 days. After the heat treatment two out of 50 eggs of *D. napolovi* hatched after 207 and 219 days (213.0 ± 0.3 days). Not all but more *D. napolovi* eggs than those of *C. morosus* stirred with stones were visually intact after the mechanical treatment and 10 eggs hatched (20%). The mean incubation time of these *D. napolovi* eggs was 200.0 ± 0.7 days. Similar to the previous species, none of the *D. napolovi* eggs treated with either acidic treatment or all factors combined hatched. The effect of treatment was also statistically significant for *D. napolovi* (*χ*^*2*^ = 121.081, d.f. = 4, *p* ≤ 0.001).

Compared to the control groups, both species showed longer incubation times in the treatment groups as well as a delayed onset of hatching.

**Fig. 5:**
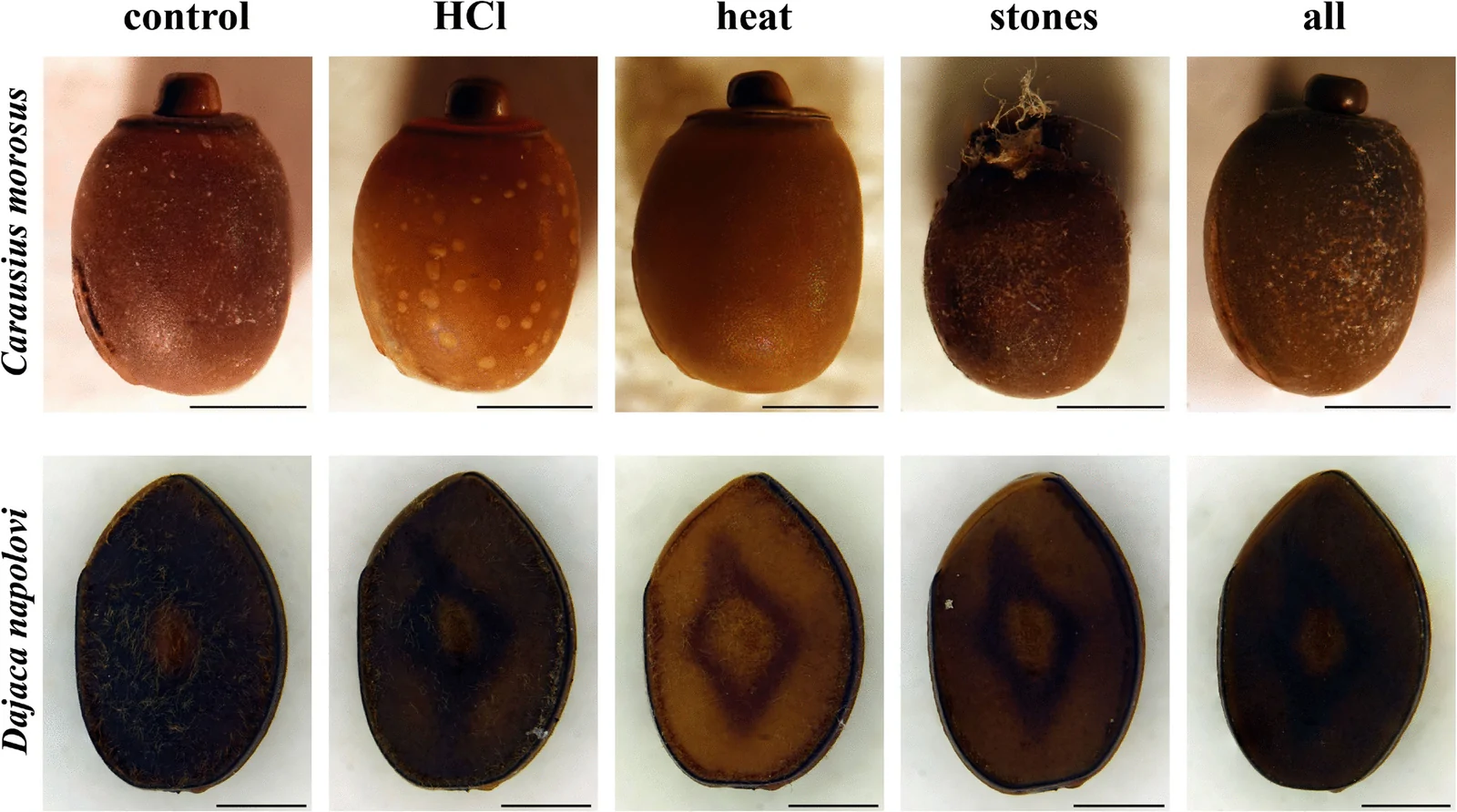
Images of treated eggs of *C. morosus* and *D. napolovi* taken via light microscope. Scale bar: 1 mm

The visual inspection of the eggs with light microscopy (Fig. 5) showed some differences between the emulated conditions of the digestive system of birds. Most visible were scratches on the eggs of *C. morosus* and loss of hair in *D. napolovi* eggs due to abrasion by the stones and all-factor treatment. Some surface changes in the form of bubble-like inflations of the chorion were found on some of the *C. morosus* eggs treated with HCl (Fig. 5). Through the inspection with the light microscope, no major changes in the appearance of eggs of *D. napolovi* treated with HCl or heat were visible.

**Fig. 6:**
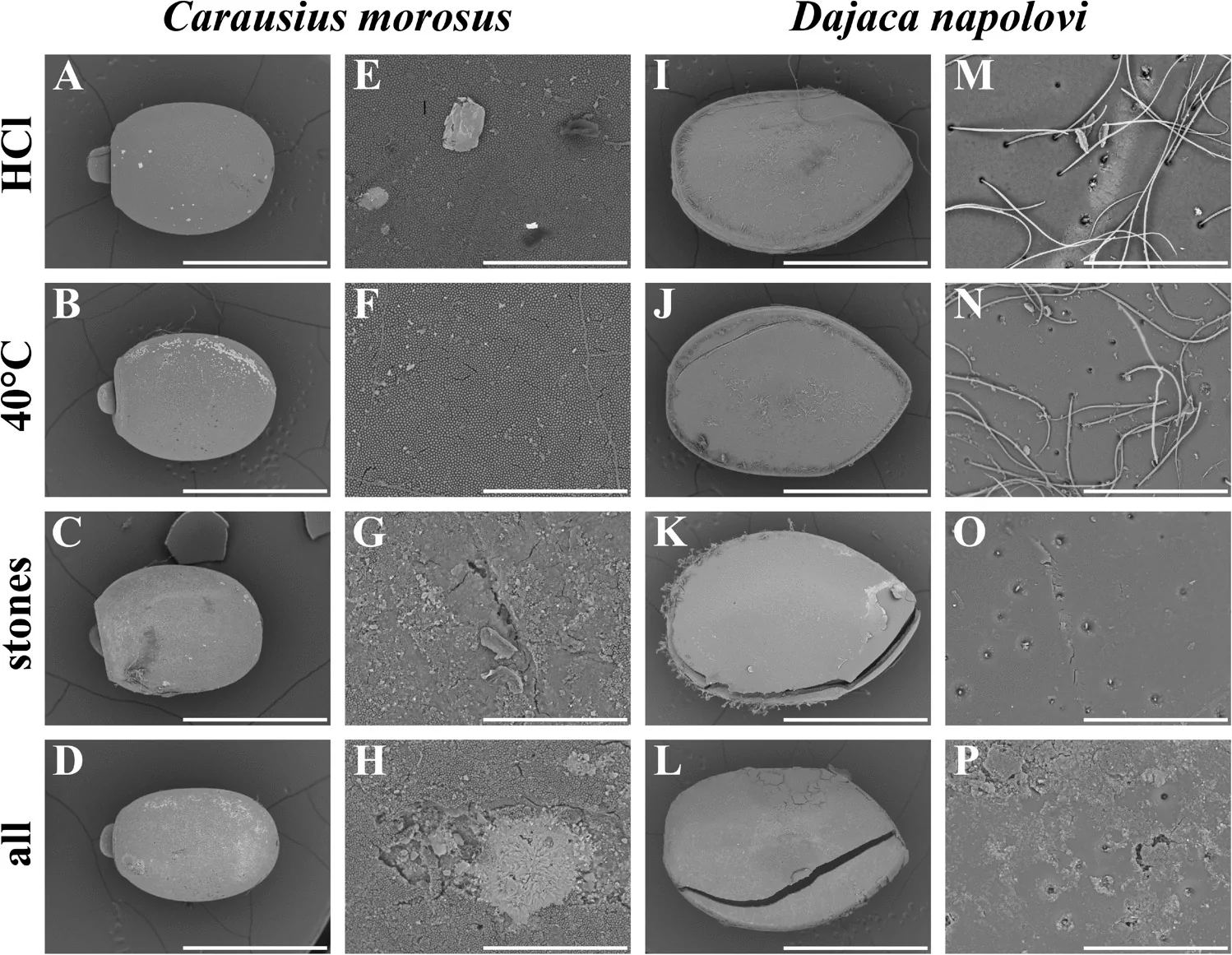
SEM images of treated *C. morosus* and *D. napolovi* eggs. **A**–**D** *C. morosus* eggs, lateral view. **E** and **F** The chorion surface of the eggs of *C. morosus*. **I**–**L** *D. napolovi* eggs, lateral view. **M**–**P** The chorion surface of the eggs of *D. napolovi*. Scale bars: **A**–**D** and **I–L**: 2 mm, **E**–**H** and **M**–**P**: 100 µm

The inspection using scanning electron microscopy (SEM) showed different changes at the surface of the eggs due to the treatments (Fig. 6). The eggs of *C. morosus* that were in contact with HCl were visibly impacted by the treatment. The chorion surface showed holes and structural damage in form of partial dissolution of the egg (Fig. 6A and B). Sometimes crystalline precipitates, probably calcium crystals that formed after dissolution from the chorion, were found on the outer layer. The surface of the eggs of *D. napolovi* that encountered a low pH occasionally revealed signs of dissolution of the chorion as well (see supplementary Fig. 1C), but these were less pronounced than in the eggs of *C. morosus*.

After the heat treatment, the eggs of *C. morosus* showed no visible changes that could be associated with high temperature. The eggs of *D. napolovi* revealed some alterations which resulted in small folds and bubbles on the surface within the shell layers. For both species scratches and cracks were found after the mechanical treatment (Fig. 6G and O; see supplementary Fig. 2). The hairs on the eggs of *D. napolovi* were scraped off in this treatment (Fig. 6O). In the all-treatment groups, the small stones also impacted the surface of the eggs and showed similar effects as in the stone treatment (Fig. 6H and P). Furthermore, some dissolution traces and the presence of crystals were found on the egg surfaces (see supplementary Fig. 1).

### Friction performance

**Fig. 7:**
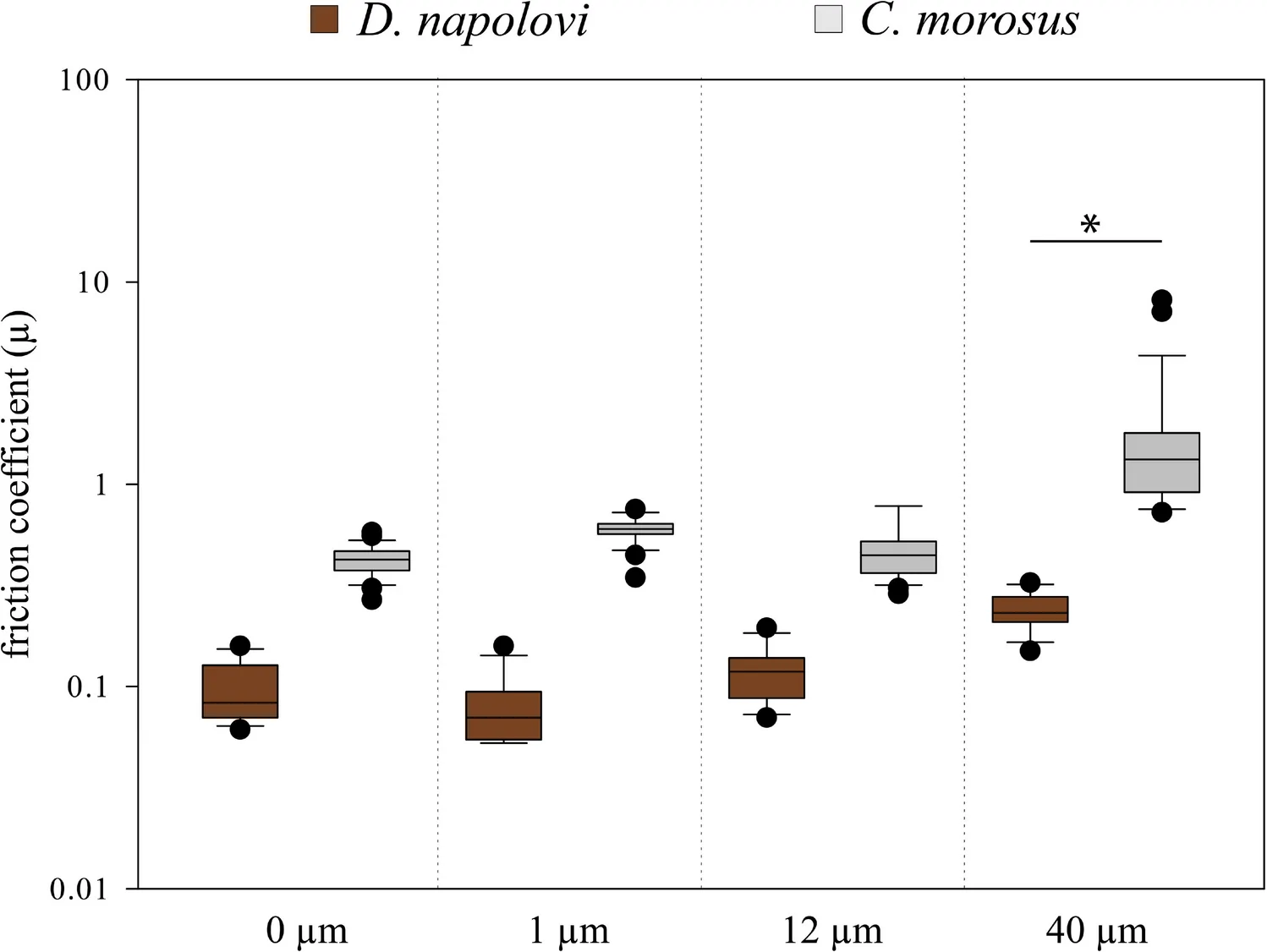
Friction coefficients of *C. morosus* and *D. napolovi* eggs on surfaces with different roughness. Statistically significant differences are marked with a * within the same roughness (Holm-Šídák post hoc test, *p* ≤ 0.001). The boxes indicate 25th and 75th percentiles, the line within the boxes represents the median, and whiskers (error bars) define the 10th and 90th percentiles

The friction coefficients (*µ*) varied across the tested substrates in both species (Fig. 7). There was a statistically significant interaction between the independent variables species and roughness (Generalized Linear Model, *F* = 5.021, d.f. = 3, *p* = 0.002). The effect of species on *µ* consequently depended on the roughness of the substrate. However, the friction coefficients were generally higher for both species on the rougher substrates (Fig. 7). On each substrate, the onset of sliding of the eggs was at higher angles for *C. morosus* compared to *D. napolovi*. Overall, the friction coefficient of *C. morosus* eggs was significantly higher compared to *D. napolovi* (Generalized Linear Model, *F* = 27.193, d.f. = 1, *p* ≤ 0.001), due to the higher sliding angles on the substrates with a roughness of 0 µm (*C. morosus*: 22.8 ± 3.6°; *D. napolovi*: 5.3 ± 1.9°), 1 µm (*C. morosus*: 30.8 ± 3.8°; *D. napolovi*: 4.6 ± 1.7°), 12 µm (*C. morosus*: 27.0 ± 14.0°; *D. napolovi*: 6.7 ± 2.0°) and 40 µm (*C. morosus*: 53.0 ± 13.3°; *D. napolovi*: 13.5 ± 2.6°). The raw data for the sliding angles and resulting friction coefficients is included in supplementary Table 2.

## Discussion

### Survival of bird passage

Previous studies have already provided insights into the potential survival of phasmids passing through a bird's digestive system and the associated distribution of these survivors (Suetsugu et al. 2018, 2023; Shelomi 2011). In Shelomi (2011) (Shelomi 2011), over 900 eggs from three different phasmid species were fed to granivorous birds, and only one unbroken egg was found in the bird droppings. Due to the mechanical effects of the beak and/or the gizzard of these granivorous birds, he concluded that dispersal via these birds is highly unlikely (Shelomi 2011). Suetsugu et al. (Suetsugu et al. 2018) presented over 200 eggs from three different species to bulbuls, common predatory birds of phasmids. Depending on the species, five to twenty percent of the eggs remained intact after passing through the bird's digestive system. Two eggs of *Ramulus mikado*, the species with the highest rate of intact eggs, survived the digestive process and hatched within the study. Consequently, they concluded that successful dispersal of phasmids through insectivorous birds remains plausible, while dispersal via granivorous birds appears unlikely (Suetsugu et al. 2018, 2023).

In contrast to the previous studies, here eggs from different species representing a wide range of different egg morphologies (O’Hanlon et al. 2020; Büscher et al. 2024; Boisseau and Woods 2024; Sellick 1997) were fed to granivorous birds and tested in the laboratory. Across all morphotypes, digestion by birds was highly destructive, and none of the eggs remained viable afterward. The survival of phasmid eggs passing through a granivorous bird's digestive system is influenced by their resistance to an acidic environment, heat, and abrasion. The extent of these factors' impact on the eggs depends on the egg shape and resistance, and consequently on the phasmid species as the phasmid egg morphology is diverse and species-specific (Brock and Büscher, 2022). The combination of all factors, and particularly a low pH value, was most influential on the eggs of phasmid species studied. Eight out of 50 *D. napolovi* eggs remained visually intact after passing through a granivorous bird within the feeding experiment of this study. This may be due to their shape, as *D. napolovi* eggs were the only disc-shaped eggs in this study, and they revealed pretty low friction coefficients on all tested substrates. This slipperiness potentially avoided crushing within the gizzard, as the eggs might slip between the stones. No intact eggs from other species were found, indicating that their eggs were crushed by the pigeons' digestive systems. None of the eggs that survived bird passage finally hatched stressing the importance of the remaining factors an egg is exposed to within the bird’s digestive system beyond mechanical destruction. For granivorous birds, the mechanical intactness of the eggs of the phamid species tested herein appears to be mainly a result of the mechanical and frictional properties of the egg chorion. No species, besides *D. napolovi*, whose eggs might be sufficiently mechanically stable to withstand the gizzard with support of their low friction coefficient, remained intact after bird passage. It is possible that eggs of other phasmid species might be similarly tough and/or slippery to mechanically overcome the gizzard of granivorous birds that ingest eggs directly. However, the comparably higher success of eggs to resist the mechanical component of the digestion of frugivorous or insectivorous birds (Suetsugu et al. 2018), especially if gravid female phasmids are ingested, might render those birds a possible vector for stick insect dispersal. Future experiments could investigate the potential of this route for rare dispersal in a similar experimental framework.

### Influence on hatching success

We herein examined the factors that ultimately affect survival of the embryo and potentially limit the dispersal capability through ornithochory. Determining factors negatively influencing the survival of the embryo sheds light on the necessary preadaptations the eggs require to establish a new population after dispersal and hatching of a parthenogenetic female (Suetsugu et al. 2023). The tested acidic environment had the greatest impact of all factors of the pigeon's digestive system on the eggs of *C. morosus* and *D. napolovi*. None of the treated eggs from both species survived the pH value around 2. The HCl most likely penetrates through the outer layers of the egg and causes significant damage. The calcium salt containing layers of the chorion are vulnerable to low pH, as strong acids react with the mineralised parts of the eggshell (Moscona 1950; Leuzinger et al. 1926; Debruyn et al. 2025). It has been recently shown that acid exposure dissolves parts of the chorion, especially in the middle layer that largely consists of calcium oxalate, and affects its mechanical properties (Reck et al. 2025). The partial dissolution of the chorion appears to be lethal for the tested eggs in this study. Acidic environment seemingly influences the normal embryo development in other insect species, too. For example, in mayflies no successful hatches occurred after being exposed to a pH of 3.0, due to its impact on the embryo or nymph (Tabak and Gibbs 1991). In another study with mosquitoes, the embryonic development was influenced and eclosion failed at a pH value of 4.0 (Aminuwa Abuh et al. 2018). The low pH of 2 in this study also affected embryonic development of the phasmid after penetrating the egg layers and was lethal.

The heat treatment seemed to have different impact on the eggs of the two species. While it appeared to strongly influence *D. napolovi*, most *C. morosus* eggs still hatched. Generally, high temperatures cause damage to proteins within the eggs, which impacts the eggs survival (Jia et al. 2020; Potter et al. 2009; Zhang et al. 2014). Considering that *C. morosus* is mainly distributed in India (Brock et al. 2025), where temperatures range between 22 °C and 35 °C during the pre-monsoon period (India meteorological department 2025), the eggs are likely adapted to withstand higher temperatures. Conversely, *D. napolovi* is found in mountainous areas in Northern Vietnam (Brock et al. 2025), where typical temperatures vary from 12.8 °C to 27.7 °C (Institute of Strategy and Policy on natural resources and environment 2009).

The mechanical influence of a granivorous bird's stomach, tested through treatment with small stones, demonstrated that crushing is a primary reason why eggs of some species do not survive being ingested by birds. However, if the eggs were not crushed, the mechanical stress and scratches on their surface did not have a significant impact. The risk of crushing seemed to depend on the shape and frictional properties of the eggs. While most *C. morosus* eggs were crushed, more *D. napolovi* eggs resisted the stone treatment. Additionally, the only visually intact eggs that withstood the bird feeding experiment were those of *D. napolovi*. Potentially, the mechanical component of birds with other dietary preferences, especially insectivorous birds that prey on phasmids, has less detrimental impact on the ingested eggs (Suetsugu et al. 2018).

In the combined treatment experiment, none of the *D. napolovi* eggs survived, whereas some *C. morosus* eggs even hatched. This may be because, when stirred together with the small stones, but not constrained in a pressurized pocket such as the bird gizzard, the eggs could move more freely across the mixture, potentially floating at the surface and therefore being not fully penetrated by the HCl solution.

The time, the eggs needed to incubate, was extended in all treatments. This was done due to the fact that in their natural environment, phasmid eggs can enter diapause to protect embryonic development when harsh conditions are present (Bedford 1978). The factors applied to the eggs could have had a similar influence and thus might lead to their prolonged incubation, especially under heat conditions (Denlinger 2001; Hodek and Hodková 1988). Mechanical stress might also induce diapause, but evidence for mechanical sensing of phasmid eggs is lacking.

In general, the dispersal through granivorous birds may be possible for phasmid eggs in rare cases, but seems to be highly unlikely. We identified three factors that prohibit successful ornithochory for the eggs: i) mechanical, ii) chemical and iii) thermal impact. Successful dispersal is possible only if embryos can cope with all three factors. Sufficient mechanical resistance was not present in any of the species tested, but slippery eggs might resist mechanical damage due to the action of the gizzard. It is possible, that some other phasmid species have eggs that are sufficiently tough to survive the gizzard of some birds (Suetsugu et al. 2018, 2023). Chemical resistance against acid influence would be required to survive the low pH regime within the birds’ digestive systems. So far there is no evidence for suitable protective egg covering, but the eggshell morphology varies between phasmid eggs and there is a chance of suitable protective proteins in the outer chorion layer (Debruyn et al. 2025). As shown herein, temperature influence can be survived, if the eggs are preadapted to higher temperatures. The extent of mechanical stress caused by the gizzard and the low pH value of the digestive systems might also be variable across bird species (Ziswiler et al. 1972) and potentially less drastic in some insectivorous and granivorous birds. According to Suetsugu et al. (Suetsugu et al. 2018), there is a chance of survival, if the eggs are sheltered inside a female phasmid that gets eaten. These phasmids must be fully grown, fertile adults from parthenogenetic species that carry mature eggs in the right timing. This renders the distribution through birds an improbable side effect rather than an evolutionary strategy. It would also only involve dispersal through insectivorous or omnivorous birds, not through granivorous birds mistaking the phasmid eggs for seeds, and thus not related to the visual seed-like appearance of the eggs. We show that the impact of the gizzard, corroborating the findings of Shelomi (2011) (Shelomi 2011), is fatal to the mechanical integrity of eggs in most tested phasmid species. However, the pH conditions in the digestive tract of granivorous birds are even more detrimental for the survival of the embryo. Future studies examining the digestive physiology of frugivorous and insectivorous birds, as well as the influence of ingested female phasmids protecting the eggs, may provide further details into how avian diet affects the potential for rare long-distance dispersal in phasmids as suggested for *R. mikado* (Suetsugu et al. 2018).

## Conclusion

The tested eggs did not pass through the granivorous birds' digestive system without damage. Although some of the *D. napolovi* eggs appeared unbroken within the bird droppings, none of them hatched. Further testing of the influence of the parameters of the birds' digestive system on the eggs showed that a low pH environment has a crucial impact on the eggs. No eggs of the two species survived the treatment with HCl concentration similar to the bird digestive system. The mechanical impact was greater for *C. morosus*, but present for both species. In addition to mechanical resistance, an egg surface with low friction (*D. napolovi*) can improve the likelihood of resisting the mechanical abrasion of the gizzard. Higher temperatures, on the other hand, had critical effects for *D. napolovi*. Overall, rare dispersal via frugivorous or insectivorous birds after predation on gravid females might be possible, but dispersal of phasmid eggs via granivorous birds is highly unlikely. Ornithochory of single eggs ingested bygranivorous birds would require a combination of mechanical resistance, chemical protection and tolerance to high temperatures. The extent to which gizzard morphology, body temperature and gastric acidity in suitable insectivorous candidate birds differ from granivorous birds and, combined with the ingestion of whole gravid phasmids sheltering fertilized eggs, might contribute to rare long-distance dispersal events in phasmids remains a subject for future study.

## Supplementary Information

Below is the link to the electronic supplementary material.

Supplementary file1 (PDF 473 KB)

Supplementary file2 (XLSX 10 KB)

Supplementary file3 (XLSX 11 KB)

## Data Availability

All raw data and additional supplementary figures are provided as electronic supplementary materials.

## Declarations

The authors used Grammarly (14.1282.0) for English grammar correction during manuscript preparation. All suggested edits were reviewed and approved by the authors, who take full responsibility for the final content.
