# Supplementary material for: Can granivorous birds disperse stick and leaf insect eggs? Integrating in vivo digestion trials and in vitro functional experiments
Source: Naturwissenschaften. 2026 Sep 11;113(5):106. doi: 10.1007/s00114-026-02157-5 (PMC13569501; doi:10.1007/s00114-026-02157-5)
Supplement: Supplementary file 1 [file 114_2026_2157_MOESM1_ESM.pdf]

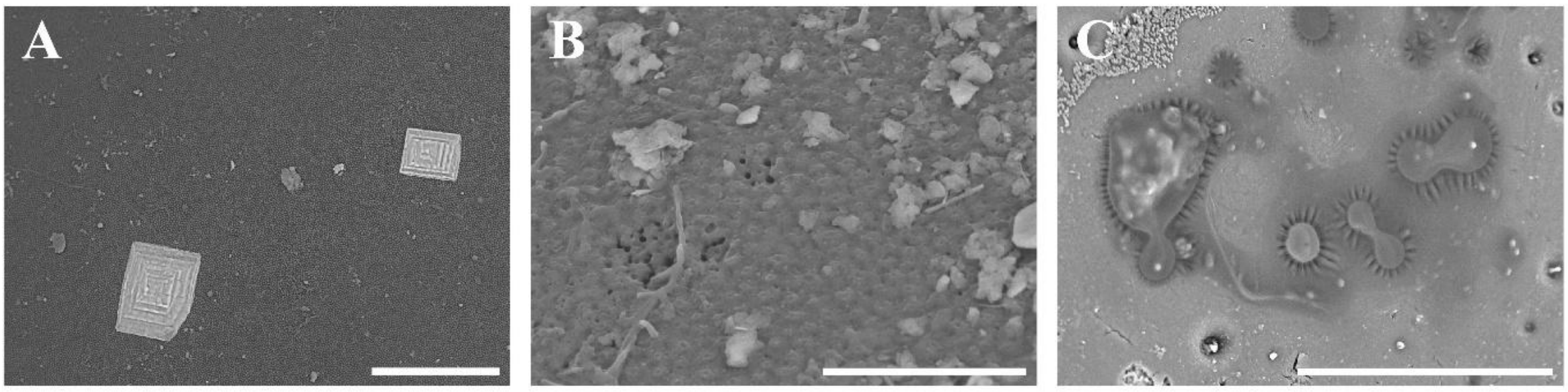

Supplementary Figure 1: SEM images of calcium crystal precipitates and surface alterations after treatment. A, B: *C. morosus*, HCl treatment; C: *D. napolovi*, all-treatment. Scale bars: A, C: 100 µm, B: 20 µm.

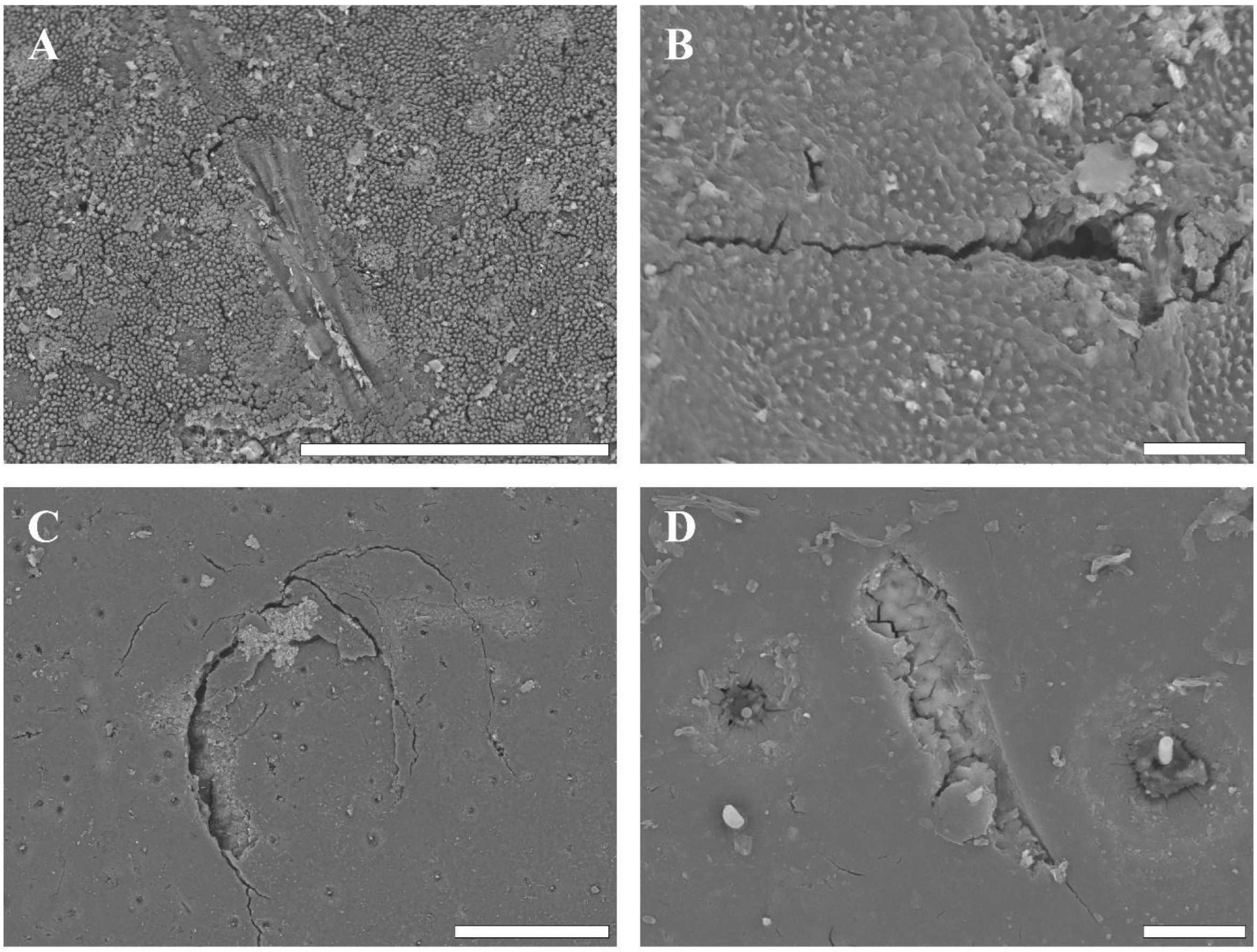

Supplementary Figure 2: SEM close-ups of mechanical impacts found on treated eggs of *C. morosus* and *D. napolovi*. A: *C. morosus* egg treated with all conditions. B: *C. morosus* egg treated with small stones. C: *D. napolovi* eggs treated with the combined set of factors. D: *D. napolovi* egg treated with small stones. Scale bars: A, D: 100 µm, B, C: 10 µm.
